# Constructing a nomogram based on the distribution of thyroid nodules and suspicious lateral cervical lymph nodes in fine-needle aspiration biopsies to predict metastasis in papillary thyroid carcinoma

**DOI:** 10.3389/fendo.2023.1242061

**Published:** 2023-11-28

**Authors:** Shui-Qing Liu, Jia-Wei Feng, Zhan-Tao Yan, Xiao-Xiao Xing, Wen-Yin Jiang, Yong Jiang, Feng Qian, Wei Xing

**Affiliations:** ^1^ Department of Ultrasound, The Third Affiliated Hospital of Soochow University, Changzhou First People’s Hospital, Changzhou, Jiangsu, China; ^2^ Department of Thyroid Surgery, The Third Affiliated Hospital of Soochow University, Changzhou First People’s Hospital, Changzhou, China; ^3^ Department of Pathology, The Third Affiliated Hospital of Soochow University, Changzhou First People’s Hospital, Changzhou, Jiangsu, China; ^4^ Department of Breast Surgery, The Third Affiliated Hospital of Soochow University, Changzhou First People’s Hospital, Changzhou, Jiangsu, China; ^5^ Department of Medical Imaging, The Third Affiliated Hospital of Suzhou University, Changzhou First People’s Hospital, Changzhou, Jiangsu, China

**Keywords:** thyroglobulin in fine-needle aspiration, lateral lymph node metastasis, lateral neck dissection, nomogram, papillary thyroid carcinoma

## Abstract

**Purpose:**

Elevated concentrations of thyroglobulin eluent is a risk factor for lateral cervical lymph node metastasis (LLNM) in patients with papillary thyroid cancer (PTC). We aimed to develop a practical nomogram based on the distribution of thyroid nodules and the presence of suspicious lateral cervical lymph nodes in fine-needle aspiration biopsies (LN-FNABs), including the cytopathology and the suspicious lateral cervical lymph node (LLN) thyroglobulin eluent (Tg), to predict the possibility of LLNM preoperatively in patients with PTC.

**Methods:**

The clinical data of PTC patients who were admitted to the Third Affiliated Hospital of Soochow University from January 2022 to May 2023 to undergo fine-needle aspiration biopsy (FNAB) were included in this study. A total of 208 patients in 2022 served as the training set (70%), and 89 patients in 2023 served as the validation set (30%). The clinical characteristics and LN-FNAB results were collected to determine the risk factors of LLNM. A preoperative nomogram was developed for predicting LLNM based on the results of the univariate and multivariate analyses. Internal calibration, external calibration, and decision curve analysis (DCA) were performed for these models.

**Results:**

The multivariate logistic regression analysis showed that the maximum thyroid nodule diameter (Odds Ratio (OR) 2.323, 95% CI 1.383 to 3.904; *p* = 0.001), Tg level (OR 1.007, 95% CI 1.005 to 1.009; *p* = 0.000), Tg divided by serum thyroglobulin, (Tg/sTg) [odds ratio (OR) 1.005, 95% CI 1.001 to 1.008; *p* = 0.009], and cytopathology (OR 9.738, 95% CI 3.678 to 25.783; *p* = 0.000) (all *p* <  0.05) had a significant impact on the LLNM of patients with suspicious LLNs. The nomogram showed a better predictive value in both the training cohort [area under the curve, (AUC) 0.937, 95% CI 0.895 to 0.966] and the validation cohort (AUC 0.957, 95% CI 0.892 to 0.989). The nomogram also showed excellent internal and external calibration in predicting LLNM. According to the DCA, the diagnostic performance of this model was dependent on the following variables: maximum thyroid nodule diameter, Tg level, Tg/sTg, and cytopathology.

**Conclusion:**

Based on the aforementioned risk factors, we believe that it is necessary to establish a personalized LLNM model for patients with PTC. Using this practical nomogram, which combines clinical and Tg risk factors, surgeons could accurately predict the possibility of LLNM preoperatively. The nomogram will also help surgeons to establish personalized treatment plans before surgery.

## Introduction

Papillary thyroid carcinoma (PTC) is endocrinology’s most common malignant tumor (1). Although PTC has an indolent nature and a good prognosis, it is also associated with an incidence of 10% to 30% in lateral lymph node metastasis (LLNM) (2, 3). Lateral cervical lymph node dissection (LND) is the main method for treating LLNM. Compared with central neck dissection, LND has some severe complications, such as nerve injury, hyperparathyroidism, chyle leakage, and neck and shoulder pain (4). Therefore, the evaluation of preoperative LLNM needs to be carried out with great caution. The cytopathology of suspicious lateral cervical lymph nodes (LLNs) in fine-needle aspiration biopsies (FNABs) can be used to clarify the status of lymph nodes (5), but the false-negative rate of cytopathology is up to 20% (6). Therefore, some scholars (7) recommend combining cytopathology with thyroglobulin to reduce this false-negative rate.

Not only the malignancy of thyroid nodules but also the distribution of thyroid nodules were related to the occurrence of LLNM in PTC. Some studies suggest that nodules located at the upper pole of the thyroid gland are more prone to LLNM (8).

In order to improve the true-positive rate and enable surgeons to have more confidence in formulating surgical plans, we developed a nomogram based on the distribution of thyroid nodules and the LN-FNAB.

## Materials and methods

### Patients

A retrospective study was conducted at the Third Affiliated Hospital of Soochow University between January 2022 and May 2023. The Institutional Review Board of Changzhou First People’s Hospital approved this study. Our study collected data from 387 patients admitted to our hospital’s thyroid surgery department, who were confirmed to have PTC through pathology and who underwent LND. All patients with suspicious lymph nodes were randomly split into the training dataset and the validation dataset (2: 1). Subsequently, based on the pathology of LND, those in the training dataset (208 patients) were divided into the LLNM group and the non-LLNM group. These patients underwent preoperative ultrasound (US) examination of the thyroid and suspicious lateral cervical lymph nodes, as well as fine-needle aspiration biopsy (LN-FNAB) for suspected lymph nodes. This retrospective study was approved by the Institutional Review Board of Changzhou First People’s Hospital, and the informed consent was waived.

**Figure 1:**
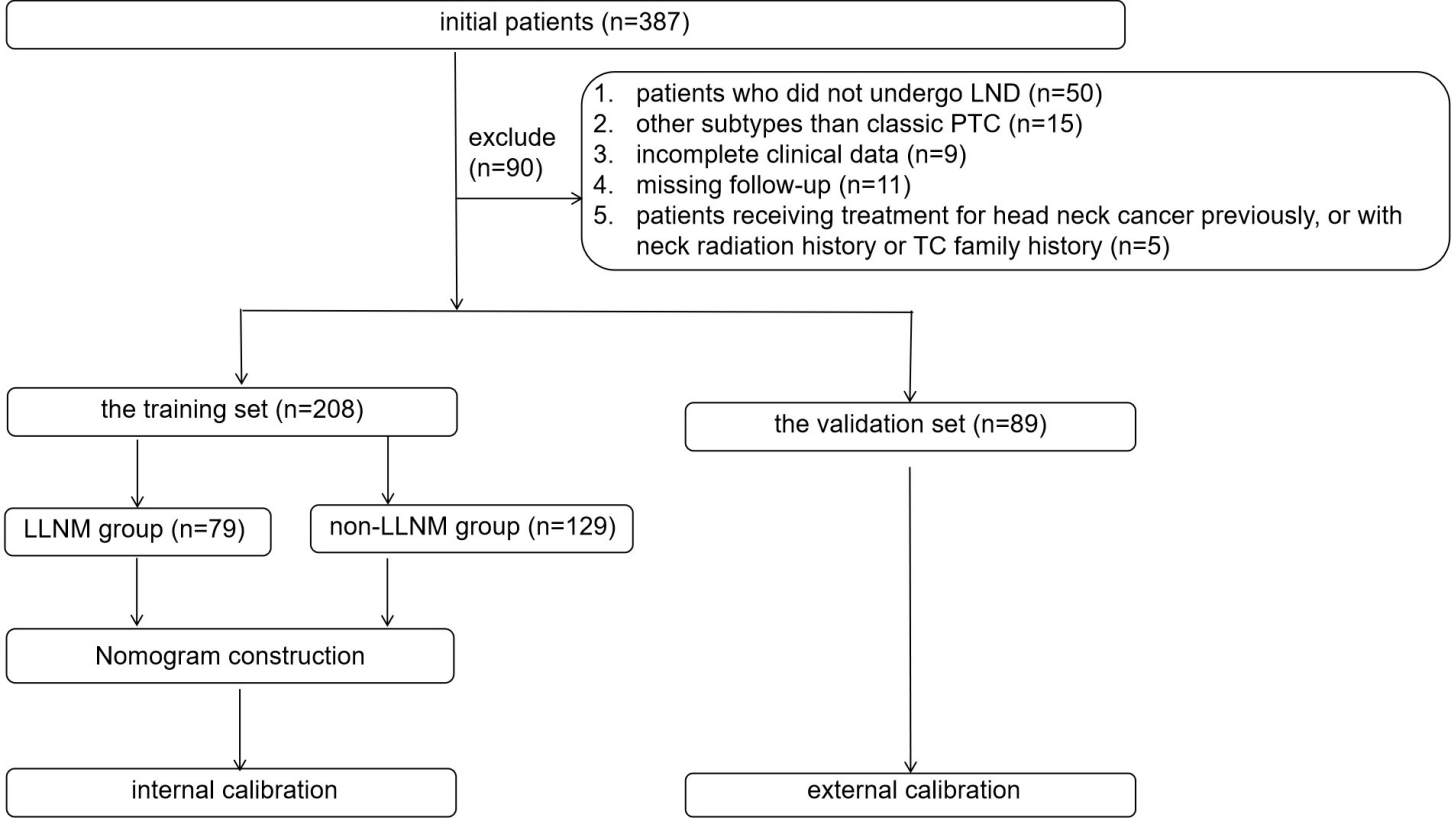
shows the flow chart of the patients enrolled in our study.

All study patients had undergone clinical examination, preoperative ultrasound, and the measurement of thyroglobulin in FNAB. The inclusion criteria were as follows: (1) patients with pathology-confirmed PTC who had underwent LND; (2) suspicious LLNs with preoperative LN-FNAB, including cytopathology and thyroglobulin; (3) complete clinical data; and (4) patients with a postoperative follow-up period of at least 6 months. The exclusion criteria were as follows: (1) patients who did not undergo LND; (2) LLNs without preoperative LN-FNABs; (3) subtypes other than classic PTC; (4) incomplete clinical data; (5) missing follow-up data; and (6) patients who had received treatment for head and neck cancer previously, or with a history of neck radiation history or a family history of thyroid carcinoma (TC). Finally, 208 patients with suspected LLNs with LND in 2022 were enrolled in this study as the training group, and 89 consecutive suspicious LLNs in 2023 were enrolled in this study as the validation group. In the training group, according to the pathological results of the LND, patients were divided into the LLNM group (*n* = 79) and the non-LLNM group (*n* = 129). **Figure 1** shows the flow chart of the patients enrolled in our study.

### Preoperative examination of thyroid nodules

The clinical and ultrasound parameters included the patients’ sex and age, the aspect ratio of the thyroid nodule (A/T), chronic lymphocytic thyroiditis (CLT), thyroid nodule site (upper pole, middle-upper third of the thyroid gland, middle pole, middle-lower third of the thyroid gland, and lower pole). If there were multiple nodules in the thyroid gland, we selected the largest or most typical nodule.

### Preoperative examination of suspicious lateral cervical lymph node thyroglobulin eluent in fine-needle aspiration biopsies

The clinical parameters included the cytopathology of the suspicious LLNs in FNABs, Tg level, and Tg/sTg. The suspicious LLNs may have one of the following ultrasound (US) characteristics, such as an A/T > 1, a globular hyperechoic mass, loss of the fatty hilum, microcalcification, and necrosis. The positive aspect of a suspicious LLN cytopathology was inherent to the detection of thyroid cells among the lymphocytes. The negative aspect of the suspicious LLN cytopathology was inherent to the fact that no thyroid cells appeared among the lymphocytes and that no suspicious LLNs were found even after 6 months of follow-up. The normal level of serum Tg ranged from 3.5 ng/mL to 77 ng/mL. Before our study, considering that the cutoff values of Tg had not been standardized, the surgeon performed LND on the basis of standard serum Tg levels. The experienced radiologists performed all the preoperative US and FNABs under ultrasound guidance. The decision of whether or not to perform LND depends on the LN-FNAB results, Tg range, doctor’s experience, and individual patient conditions. (1) When the cytopathology revealed definite malignant cells in the suspicious LLNs and the Tg level exceeded 77 ng/mL, the surgeon performed LND; (2) when indefinite malignant cells were found in the cytopathology or the Tg level ranged from 3.5 ng/mL to 77 ng/mL, LND was carefully considered; (3) if both the cytopathology or the Tg less than 3.5ng/mL, surgeons would carry out preventive lateral neck LND based on their own experience and individual circumstances; and (4) if both the cytopathology and the Tg were negative, and there were no surgical indications based on the experience of the physician and the individual condition of the patient, then LND was avoided, but it was suggested that patients were followed up at least every 3 months. Considering the fact that some patients have a high level of serum Tg, which could have been an indication of nodular goiter or subacute thyroiditis, some studies (9) suggested incorporating Tg/sTg into the study.

### Surgery and pathology

As suspicious lateral cervical lymph nodes were discovered in patients with thyroid nodules, a detailed preoperative examination including FNAB and clinical characteristics were carried out. We determined the status of suspicious LLNs based on the results of cytopathology and the level of Tg before surgery. The LND was performed for patients with a positive FNAB. The resected thyroid and LLN specimens were processed for pathological examination (including evaluating for the existence of chronic lymphocytic thyroiditis, maximum size of the thyroid nodule, and the neck level of the metastatic nodes). The pathological results pertaining to the thyroid nodules and LLNs were used as the gold standard.

### Pathological examination

Two or more experienced pathologists, who were blinded to the clinical characteristics and ultrasonographic findings, reviewed all the surgical specimens. The gold standard of this study was the pathological diagnosis. Every patient who needed to undergo LND was marked on the skin under preoperative ultrasound guidance and injected with tissue glue around the suspected lymph nodes. During the surgery, the surgeon would first remove the marked lymph nodes and send them to the pathology department separately. Subsequently, the lymph nodes around the suspected lymph nodes that are visible or palpable to the naked eye were also removed. Each cervical lymph node was fixed in 20% buffered formalin, embedded in paraffin, sectioned, and stained with hematoxylin and eosin. The pathologists investigated the lymph nodes with suspected cancer through immunohistochemical staining. All the pathological specimens were reviewed and cross-checked by two or more experienced pathologists microscopically.

### Statistical analyses

The statistical analysis was performed using IBM SPSS Statistics version 26.0 software (IBM Corporation, Armonk, NY, USA), MedCalc (version 15.6; MedCalc software, Mariakerke, Belgium), and R software version 3.5.3 (The R Foundation for Statistical Computing, Vienna, Austria). Normally distributed variables were expressed as means ± standard deviations (SDs), and skew-distributed variables were reported as numbers and percentages. An independent *t*-test, a Pearson’s chi-squared test, or a Fisher’s exact test was used for the univariable analysis. The variables with statistical differences (*p* < 0.05) in the univariate analysis were included in the multivariate binary logistic regression analysis to determine the independent risk factors affecting lateral cervical lymph node metastasis. These independent predictors were used to construct the nomogram in R software for predicting the risk of LLNM. We used the receiver operating characteristic (ROC) curve in MedCalc to test our established prediction model’s discriminative power and consensus. The performance of the nomogram was further evaluated using the calibration chart, which plotted the predicted probability of the nomogram against the observed probability. In the nomogram, each variable was estimated into scores, and the scores of each variable were then added for each patient to determine the probability of LLNM based on their total score. The internal calibration curve, external calibration curve, decision curve analysis (DCA), and ROC were used to evaluate the discrimination and calibration ability of the training set and the validation set. The statistical significance was set at a *p*-value < 0.05.

## Results

### Patient characteristics

**Table 1** shows the comparison of the clinical baseline data of the training set and the validation set. A total of 208 patients in 2022 entered the training set (70%), and 89 patients from January to May 2023 entered the validation set (30%). There were no significant differences between the training set and the validation set (*p* > 0.05 for all comparisons).

**Table 1:** Clinical characteristics of the training set and the validation set.

Characteristics	Training set (n=208)		Validation set (n = 89)	*P* value
	LLNM- (n=129)	LLNM+ (n=79)		
Gender				
female	84	57	60	
male	45	22	29	0.950
Age(y)				
>36	92	41	55	
<=36	37	38	34	0.948
CLT				
absence	108	62	71	
presence	21	17	18	0.694
A/T				
<=0.71	22	23	19	
>0.71	107	56	70	0.676
Maximum tumer size (cm)				
<1.1	96	25	46	
>1.1	33	54	43	0.506
Location of thyroid nodules 1				
left lobe	69	30	50	
right lobe	56	47	35	
bilateral	4	1	1	
isthmus	0	1	3	0.244
Location of thyroid nodules 2				
upper pole	31	28	21	
middle upper one-third of the thyroid gland	18	21	15	
middle pole	43	21	32	
middle lower one-third of the thyroid gland	20	4	11	
lower pole	17	5	10	0.373
Location of thyroid nodules 3				
ventral side	28	7	14	
dorsal side	19	10	17	
inside	5	3	2	
outside	9	6	5	
penetrating	51	40	39	
central	17	13	12	0.813
Location of lymph nodes				
left	72	32	50	
right	57	47	39	0.330
Level of lymph nodes				
2	12	0	7	
3	56	32	34	
4	58	38	41	
5	0	2	1	0.771
Cytopathology				
Negative	116	21	56	
Positive	13	58	33	0.627
Tg				
<61.6	107	10	52	
>61.6	22	69	37	0.605
Tg/s-tg				
<2.91	104	7	51	
>2.91	25	72	38	0.543

LLNM, lateral cervical lymph node metastasis; CLT, chronic lymphocytic thyroiditis; A/T, aspect ratio (height divided by width on transverse views); Tg, thyroglobulin in fine-needle aspiration, 61.6 is the cutoff value obtained from this study; Tg/s-tg, Tg divided by serum thyroglobulin, 2.91 is the cutoff value obtained from this study.

If there are multiple nodules in the thyroid gland, we selected the largest or most typical nodule.

The positive of cytopathology refers to the detection of thyroid cells in the background of lymphocytes. The negative of cytopathology means no thyroid cells appeared in the background of lymphocytes and no suspicious LLNs were founded after at least six months of follow-up.

P value < 0.05 indicates a significant difference between the training set and the validation set.

In our study, 387 patients with 387 suspicious LLNs underwent LLN FNAB between January 2022 and December 2022. Finally, 208 patients who underwent LND were enrolled in this study. The postoperative pathology revealed the presence of LLNM in 79 patients with suspicious LLNs (**Figure 1**).

### Distribution of suspicious lateral cervical lymph nodes in the lateral neck

As summarized in **Table 2**, of the 208 PTC patients with 208 suspicious LLNs, 67 men (32.2%) and 141 women (67.7%) underwent LND in our study, with an average age of 43.3 years ± 11.9 years (range from 18 years to 78 years), a maximum thyroid nodule diameter of 1.25 cm ± 0.9 cm (range from 0.22 cm to 6.0 cm), and an A/T of 0.8 ± 0.1 (range from 0.37 to 1.2). The suspicious LLNs in the right neck were detected in 104 (50%) patients, and tumors in the left neck were detected in 104 (50%) patients (**Table 1**).

**Table 2:** Clinical characteristics of suspicious LLNs.

Characteristics		value
Gender	female	141
	male	67
Age(y)	range	18-78
	mean+SD	43.3±11.9
CLT	absence	170
	presence	38
A/T	range	0.37 - 1.2
	Mean+SD	0.8±0.1
Maximum tumer size (cm)	range	0.22 - 6.0
	Mean+SD	1.25±0.9
Location of thyroid nodules 1	left lobe	99
	right lobe	103
	bilateral	1
	isthmus	5
Location of thyroid nodules 2	upper pole	59
	middle upper one-third of the thyroid gland	39
	middle pole	64
	middle lower one-third of the thyroid gland	24
	lower pole	22
Location of thyroid nodules 3	ventral side	35
	dorsal side	29
	inside	8
	outside	15
	penetrating	91
	central	30
Location of lymph nodes	left	104
	right	104
Level of lymph nodes	2	12
	3	88
	4	96
	5	2
	6	10
Cytopathology	Negative	137
	Positive	71
Tg	median	19.44
	IQR	10.55-500
Tg/s-tg	median	2.45
	IQR	0.08-29.11

LLNM, lateral cervical lymph node metastasis; CLT, chronic lymphocytic thyroiditis; A/T, aspect ratio (height divided by width on transverse views); Tg, thyroglobulin in fine-needle aspiration, 61.6 is the cutoff value obtained from this study; Tg/s-tg, Tg divided by serum thyroglobulin, 2.91 is the cutoff value obtained from this study.

If there are multiple nodules in the thyroid gland, we selected the largest or most typical nodule.

The positive of cytopathology refers to the detection of thyroid cells in the background of lymphocytes. The negative of cytopathology means no thyroid cells appeared in the background of lymphocytes and no suspicious LLNs were founded after at least six months of follow-up.

P value < 0.05 indicates a significant difference between the training set and the validation set.

### Risk factors for lateral cervical lymph node metastasis in papillary thyroid carcinoma patients

LLNM had a significant association with the maximum thyroid nodule diameter, tumor site, neck level of the metastatic nodes, cytopathology, Tg level, and Tg/sTg in the univariate analysis (all *p* < 0.05). A binary logistic regression equation of lateral cervical lymph node metastasis was constructed based on the variables used in the univariate analysis. The multivariate analysis showed that the maximum thyroid nodules diameter [odds ratio (OR) 2.323, 95% CI 1.383 to 3.904; *p* = 0.001], Tg level (OR 1.007, 95% CI 1.005 to 1.009; *p* = 0.000), Tg/sTg (OR 1.005, 95% CI 1.001 to 1.008; *p* = 0.009), and cytopathology (OR 9.738, 95% CI 3.678 to 25.783; *p* = 0.000) remained independent predictors for LLNM in PTC patients with suspicious LLNs (**Tables 2**–**4**).

**Table 3:** Univariate analysis of factors associated with LLNM.

Characteristics	Univariate analysis		
	LLNM group (n = 79)	non-LLNM group (n = 129)	*P* value
Gender(n)			0.359
female	57	84	
male	22	45	
Age(y)			0.468
>36	41	92	
<=36	38	37	
CLT			0.360
negative	62	108	
positive	17	21	
A/T			0.054
>=1	3	14	
<1	76	115	
Maximum tumer size (cm)	1.62±0.926	1.03±0.8	
Location of thyroid nodules 1			0.061
left lobe	30	69	
right lobe	47	56	
bilateral	1	0	
isthmus	1	4	
Location of thyroid nodules 2			0.007
upper pole	28	31	
middle upper one-third of the thyroid gland	21	18	
middle pole	21	43	
middle lower one-third of the thyroid gland	4	20	
lower pole	5	17	
Location of thyroid nodules 3			0.242
ventral side	7	28	
dorsal side	10	19	
inside	3	5	
outside	6	9	
penetrating	40	51	
central	13	17	
Location of lymph nodes 1			0.045
left	32	72	
right	47	57	
Level of lymph nodes			0.004
2	0	12	
3	32	56	
4	38	58	
5	2	0	
6	7	3	
Tg			0.000
Median (IQR)	500 (176.3-500)	2.46 (0.49-22.84)	
Tg/s-tg			0.000
Median (IQR)	34.04 (6.16-155.76)	0.25 (0.04-2.45)	
Cytopathology			0.000
Negative	21	116	
Positive	58	13	

LLNM, lateral cervical lymph node metastasis; CLT, chronic lymphocytic thyroiditis; A/T, aspect ratio (height divided by width on transverse views); Tg, thyroglobulin in fine-needle aspiration, 61.6 is the cutoff value obtained from this study; Tg/s-tg, Tg divided by serum thyroglobulin, 2.91 is the cutoff value obtained from this study.

If there are multiple nodules in the thyroid gland, we selected the largest or most typical nodule.

The positive of cytopathology refers to the detection of thyroid cells in the background of lymphocytes. The negative of cytopathology means no thyroid cells appeared in the background of lymphocytes and no suspicious LLNs were founded after at least six months of follow-up.

P value < 0.05 indicates a significant difference between the training set and the validation set.

**Table 4:** Multivariate analysis of factors associated with LLNM.

Characteristics	multivariate analysis	
	Adjusted OR (95% CI)	*P* value
Maximum tumer size (cm)	2.364 (1.985-2.810)	0.047
Location of thyroid nodules 2		
upper pole	ref	
middle upper one-third of the thyroid gland	0.408 (0.100-1.676)	0.214
middle pole	0.366 (0.112-1.194)	0.096
middle lower one-third of the thyroid gland	0.185 (0.028-1.219)	0.079
lower pole	0.146 (0.025-0.840)	0.051
Location of lymph nodes 1		
left	ref	
right	2.157 (0.859-5.420)	0.102
Tg	1.005 (1.003-1.007)	0.000
Tg/s-tg	1.005 (1.001-1.009)	0.009
Cytopathology	9.738 (3.678-25.783)	0.000

LLNM, lateral cervical lymph node metastasis; CLT, chronic lymphocytic thyroiditis; A/T, aspect ratio (height divided by width on transverse views); Tg, thyroglobulin in fine-needle aspiration, 61.6 is the cutoff value obtained from this study; Tg/s-tg, Tg divided by serum thyroglobulin, 2.91 is the cutoff value obtained from this study.

If there are multiple nodules in the thyroid gland, we selected the largest or most typical nodule.

The positive of cytopathology refers to the detection of thyroid cells in the background of lymphocytes. The negative of cytopathology means no thyroid cells appeared in the background of lymphocytes and no suspicious LLNs were founded after at least six months of follow-up.

P value < 0.05 indicates a significant difference between the training set and the validation set.

### Validation of the nomogram

**Figure 2:**
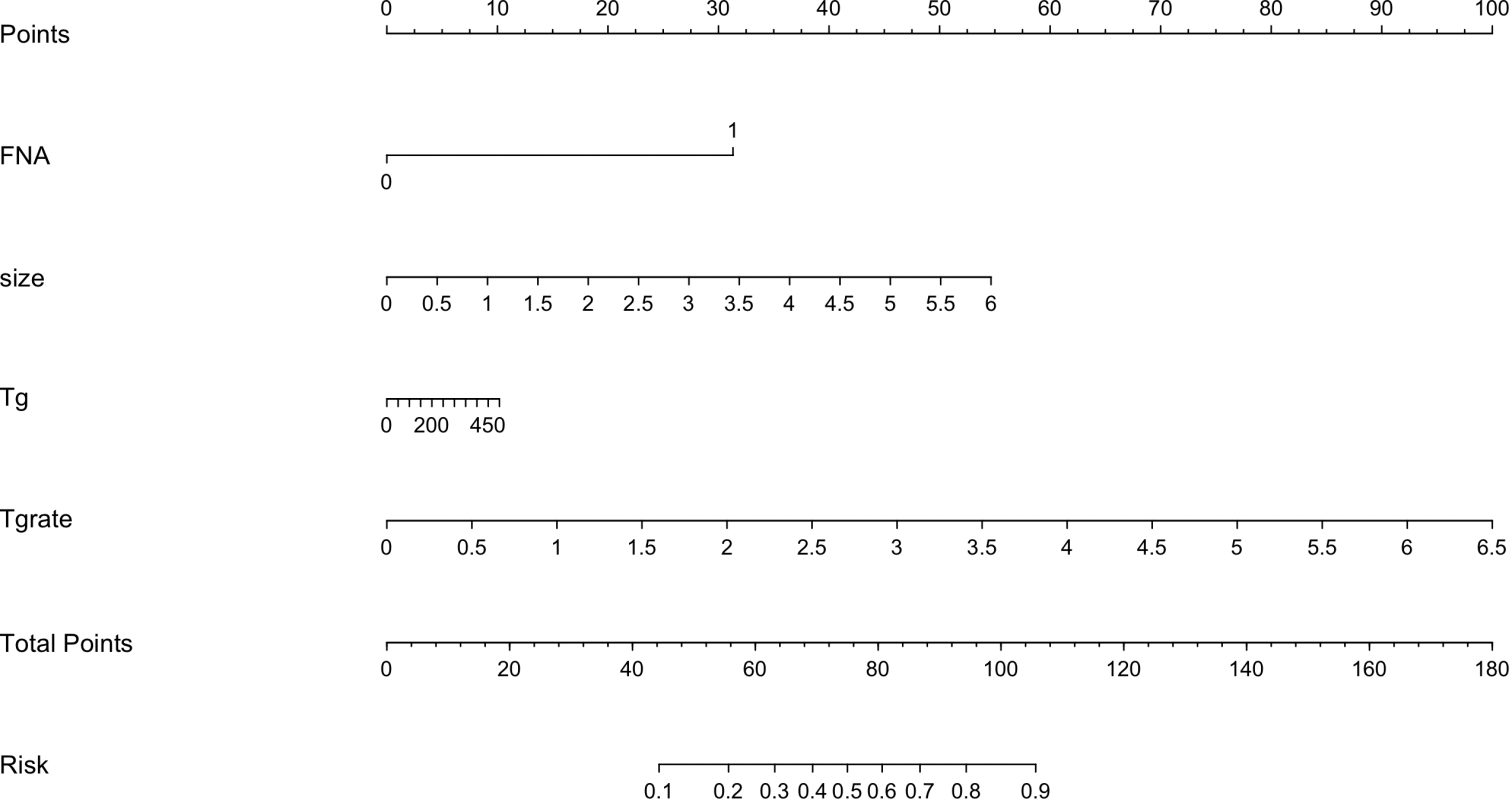
Nomogram for predicting LLNM in patients with PTC.

**Figure 3:**
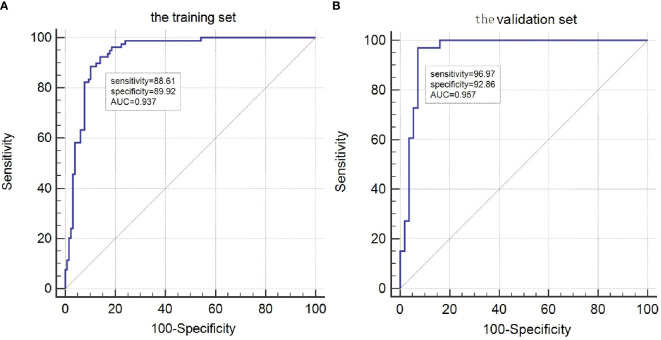
ROC curves for the LLNM model. **(A)**The ROC of the training group (AUC =0.937);**(B)** The ROC of the validation group (AUC =0.957).

**Figure 4:**
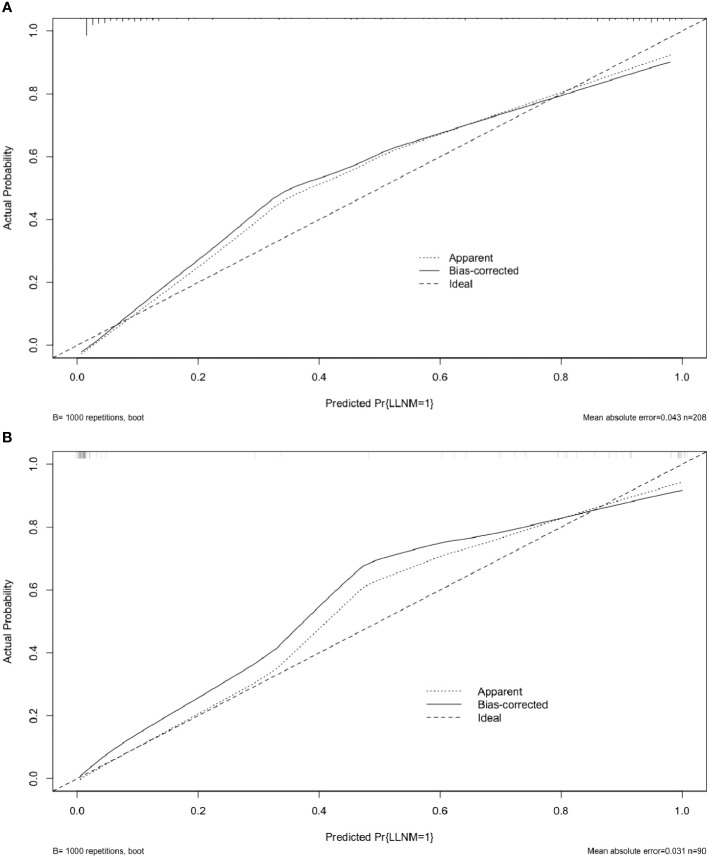
Calibration curves for the LLNM model. These are calibration curves of nomograms for predicting LLNM. The diagonal dashed line represents the ideal prediction made using the perfect nomogram; the solid line represents the calibration estimate from the internally validated model; and the dotted line indicates the apparent predictive accuracy. **(A)** The calibration curves of the training group;**(B)**The calibration curves of the validation group. The closer the solid line is to the dotted line, then the stronger the LLNM model’s predictive ability.

**Figure 5:**
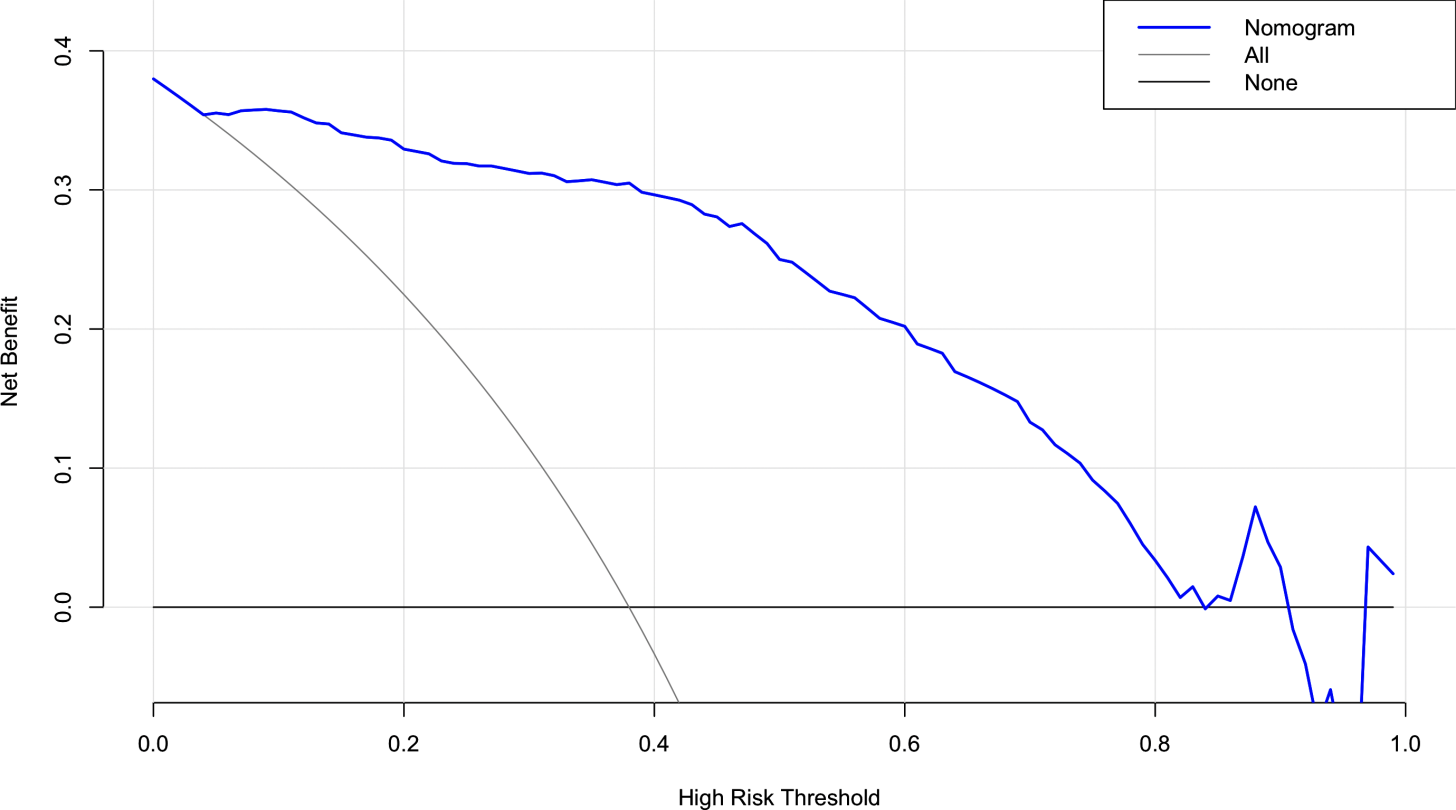
DCA for the LLNM model.

The risk factors, including maximum thyroid nodule diameter, Tg level, Tg/sTg, and cytopathology, identified through multivariate analysis were included in the nomogram for predicting LLNM. The nomogram showed that the maximum thyroid nodule diameter and cytopathology had the greatest effects on the score (**Figure 2**). Thereafter, this prediction model was used to develop the ROC analysis. The area under the curve (AUC) of the training model was 0.937 (95% CI 0.895 to 0.966), the sensitivity was 0.88, and the specificity was 0.89 (**Figure 3**). The AUC of the validation model was 0.957 (95% CI 0.892 to 0.989), the sensitivity was 0.96, and the specificity was 0.92 (**Figure 3B**). In addition, we developed the internal and external validation of the LLNM model. In the nomogram, the solid line represents the true LLNM, whereas the dotted line represents the virtual incidence rate based on the prediction model. The higher the matching degree between the dashed and solid lines, the higher the accuracy of the prediction model. Our prediction model showed that the risk of LLNM was consistent with the actual results. Furthermore, we conducted the external calibration for the established model. The AUC of the training cohort and validation cohort were in good agreement (**Figures 4A, B**). The decision curve analysis (DCA) was also frequently used to evaluate the practical value of these models (**Figure 5**). The abscissa of this graph represents the threshold probability. The vertical axis is the net benefit (NB) after subtracting the pros and cons. The solid black line (none line) and the solid grey line (all line) represent two extreme situations, respectively. The first extreme situation is represented by the solid black line. At this point, it was assumed that the patient did not have LLNM and had not undergone LND, and the NB was zero. The second extreme situation is represented by the solid grey line. At this point, it was assumed that all patients had LLNM and had undergone LND. The NB is a backslash line. The solid blue line is the result of our nomogram. The solid blue line performed significantly better than the solid grey line at most of the threshold points.

## Discussion

The presence of LLNM in PTC patients often indicates poor prognosis and local recurrence; therefore, the preoperative evaluation of LLNM was the focus of this study. According to the American Thyroid Association (ATA) guidelines, LND is necessary for PTC patients with LLNM (8). Prophylactic ipsilateral cervical LND is still a controversial topic. Its supporters have pointed out that preventive ipsilateral cervical LND could eliminate the possibility of local recurrence (6), but its opponents believe that prophylactic ipsilateral LND could increase the probability of some potential complications such as permanent hypoparathyroidism (10). At present, compared with prophylactic LND, therapeutic CND is more widely recognized by many surgeons (11, 12). Many studies have focused on evaluating the accuracy of surgery in PTC while ignoring the distribution of thyroid nodules within the thyroid gland. Many scholars have reported that the incidence of LLNM varied from 10% to 30%. However, the frequency of LLNM was 37.9% (i.e., in 79 out of 208 patients) in our study. This difference could be due to the selection bias, because the samples included in our study were those of patients with PTC.

Many clinical factors related to LLNM have been previously reported, including sex, age, CLT, and tumor size (13, 14). In addition to the clinical features mentioned above, we also added some parameters of preoperative evaluation. According to our findings, the maximum thyroid nodule diameter, tumor location, cytopathology, Tg level, and Tg/sTg were risk factors in the univariate analysis. However, in the multivariate analysis, the maximum thyroid nodule diameter, cytopathology, Tg level, and Tg/sTg were independent risk factors for LND.

All of the above factors can be used to create individual possibilities for clinical events and ultimately help clinicians in decision-making. These clinical parameters were incorporated into the nomogram as risk factors for LLNM.

As some investigators have reported, a malignant tumor has the characteristic of unlimited proliferation (15). In our study population, the cutoff value for the maximum diameter of a thyroid nodule was greater than 1.1 cm. As the maximum thyroid nodule diameter of thyroid nodules increases, the probability of LLNM also increases (16, 17). One of the influencing factors is the distribution of thyroid nodules within the thyroid gland. There are five distribution modes, namely the upper pole, middle-upper third of the thyroid gland, middle pole, middle-lower third of the thyroid gland, and the lower pole. Many studies have focused on analyzing the possibility of LLNM from the morphology of thyroid nodules (18), but have overlooked the relationship between the distribution of thyroid nodules and LLNM. Due to the special location and lymphatic drainage characteristics of the isthmus of the thyroid gland, the lymphatic vessels in the isthmus drain into the anterior larynx and anterior trachea lymph nodes, leading to a higher incidence of CLNM (9). Gong Y and Ito Y’s research (19, 20) shows that, compared with the nodules located at the middle pole of the thyroid gland, nodules at the upper pole and middle-upper third of the thyroid gland are extremely prone to LLNM. This is mainly because the lymphatic drainage system of the thyroid gland is similar to the venous drainage system (21). In our study, the prevalence of LLNM was 63.2% (i.e., in 50 out of 79 patients) in the upper pole or the upper part of the high plane of the isthmus of the thyroid gland, which was higher than the thyroid nodules in middle pole and isthmus (22.7%). Moreover, we evaluated the distribution of LLNM in the different levels of the neck. We found that the incidence of LLNM was highest in level 4 of the lateral neck (71.4%), followed by level 3 and level 5 (25% and 3.5%, respectively). The main reason for this might be related to the lymphatic drainage, which collects lymph from the upper and middle poles of the thyroid gland. Therefore, we suggest that during the preoperative evaluation, the thyroid nodules located at the upper or middle poles should be carefully evaluated for the condition of the lateral neck lymph nodes, especially those in level 4.

Another important influencing factor is the measurement of Tg concentration (22). As we all know, thyroid follicular epithelial cells secrete thyroglobulin (23, 24). ATA (25) and the European Thyroid Association (26) recommended that measuring the concentration of thyroglobulin in lymph nodes increases the accuracy of the diagnosis of LLNM (9). The key factors included the amount of Tg remaining in the needle tip and any volume of diluent. The ideal diluent should have the following characteristics (27). The first point is that it should have less influence on the conformation of proteins. Another point is that the diluent needs to be readily available. Currently, the available diluents include normal saline and Tg-free solution (28). Normal saline is more widely used than Tg-free solution, and it is reported that normal saline is a more economical option and exerts a smaller matrix effect (29, 30). Our study adopted 1 mL of normal saline as the diluent. The cutoff value of Tg has been controversial. Some scholars (31) set the cutoff value to 1 ng/mL, whereas Kim et al. (32) set the cutoff value to 10 ng/mL. The fluctuation range of this cutoff value is too extensive, leading to many false-positive and false-negative cases. In our study, the cutoff values of Tg and Tg/sTg were 61.6 ng/mL and 2.91 ng/mL (without a log conversion), and the associated sensitivity and specificity were 87.3% vs. 82.9% and 91.1% vs. 81.4%, respectively.

In the debate about prophylactic ipsilateral cervical LND with PTC, our model provides a reference for the development of surgical methods through personalized preoperative evaluation. Meanwhile, based on the model, we stratified the risk level of PTC patients based on their preoperative evaluation results. The patients at the different risk levels were treated using different strategies. In our nomogram, the low risk level refers to PTC patients with a relatively low level (< 30%) of ipsilateral LLNM, for whom prophylactic ipsilateral LND should be avoided. The moderate risk level refers to PTC patients with a moderate level (30%–70%) of ipsilateral LLNM, for whom whether or not LND should be performed needs to be carefully evaluated. The specific process was to locate the suspicious LLNs first, then remove them and send it for rapid pathology, and finally to determine whether or not to perform LND based on the results of the rapid pathology. The high risk level is PTC patients with a high level (> 70%) of ipsilateral LLNM, for whom ipsilateral LND is recommended. Therefore, our nomogram could be used carefully, and to guide surgeons in their assessment of the suspected LLN status and to enable them to avoid unnecessary LND. The nomogram usage method is as follows. First, the scores corresponding to each indicator of the patient should be recorded, and then, these scores should be added together to correspond to the risk level on the horizontal axis. Finally, the surgical method can be determined based on the risk level (14).

Although this study provides a more appropriate evaluation method, it still has some limitations. In our study, 13 patients had a positive cytopathology, but negative histopathology. We speculate that the reasons may be as follows: (1) although we provided preoperative localization, the suspected lymph nodes were small in size, concealed in location, and were difficult for surgeons to detect; (2) the puncture area of the lymph nodes was contaminated, resulting in the presence of false positives; (3) last but not least, this may have been related to the ectopic thyroid tissue, which cannot be clearly distinguished under US. Therefore, during the puncture process, fine needles were inserted into the ectopic thyroid tissue, resulting in false-positive results. Frasoldati et al. (33) believed that there was only a weak correlation between sTgAb and FNAB Tg, but Baskin et al. (34) and Boi et al. (35) believed that the correlation between them were worth discussing. In the future, we will first perform FNAB on suspected lymph nodes, and then focus on thyroid nodules. Meanwhile, during the puncture process, the puncture needle should be directly inserted into the suspected lymph nodes to avoid interference from ectopic thyroid as much as possible. Compared with other multicenter prospective studies, the patients in our study were retrospectively selected and came from only one medical center. Hence, the selective bias of the sample was inevitable. In addition, different sonographers performed the preoperative evaluation of suspicious LLNs. Due to the limited sample size, this study included only the training group and not the validation group, which will be the next work plan of this study.

In conclusion, our nomogram showed that LLNM was independently associated with the maximum of thyroid nodules, cytopathology, Tg level and Tg/sTg. It was possible to obtain the individual incidence probability for each PTC patient with suspected LLNs using the nomogram, which provides a critical reference for the clinical preparation of a surgical plan.

## Data availability statement

The original contributions presented in the study are included in the article/supplementary material. Further inquiries can be directed to the corresponding authors.

## Ethics statement

The studies involving humans were approved by The Institutional Review Board of Changzhou First People’s Hospital. The studies were conducted in accordance with the local legislation and institutional requirements. Written informed consent for participation in this study was provided by the participants’ legal guardians/next of kin.

## Author contributions

S-QL and J-WF: writing—original draft, software, and data curation. Z-TY and X-XX: validation, formal analysis, and data curation. W-YJ: conceptualization. YJ: validation and investigation. WX and FQ: writing—review and editing, visualization, and supervision. All authors contributed to the article and approved the submitted version.
